# Partial quadrate lobectomy improves early outcomes of laparoscopic Kasai surgery in type III biliary atresia

**DOI:** 10.3389/fped.2025.1541455

**Published:** 2025-05-14

**Authors:** Chunhui Gu, Jian Sun, Lihong Ding, Bing Li, Youcheng Zhang, Guoqing Jiang

**Affiliations:** ^1^ Department of Pediatric Surgery, Huai’an Maternal and Child Health Care Hospital Affiliated to Yangzhou University, Huai’an, China; ^2^ Department of Hepatobiliary Surgery, Northern Jiangsu People's Hospital, Yangzhou, China; ^3^ Department of Hepatobiliary Surgery, Northern Jiangsu People's Hospital Affiliated to Yangzhou University, Yangzhou, Jiangsu, China; ^4^ Nanjing University of Chinese Medicine, Nanjing, China

**Keywords:** laparoscopic Kasai, biliary atresia, partial quadrate lobectomy, segmentectomy, postoperative outcomes

## Abstract

**Objective:**

To evaluate the early efficacy and safety of partial quadrate lobectomy during laparoscopic Kasai surgery for type III biliary atresia.

**Methods:**

This retrospective study included 25 children diagnosed with type III biliary atresia, who underwent laparoscopic Kasai surgery between February 2018 and July 2022. Patients were divided into two groups: one with partial quadrate lobectomy and the other without. Data collected included age, gender, weight, incidence of cholangitis before and after surgery, one-year native liver survival, intraoperative blood loss, surgery duration, and jaundice clearance at 6 and 12 months. Follow-up results were compared between the groups.

**Results:**

The partial quadrate lobectomy group (14 patients) had a mean weight of 5.50 kg and average age of 66.79 days, while the control group (11 patients) had a similar weight (5.50 kg) and a mean age of 71.09 days. Weight comparison showed no significant difference (5.50 kg vs. 5.50 kg, *P* = 0.427). One-year postoperative native liver survival was 9/14 in the partial quadrate lobectomy group vs. 6/11 in the control group (*P* = 0.654).Intraoperative blood loss was similar between groups (*P* > 0.05), but the shorter operative time (301 vs. 347 min) associated with partial quadrate lobe resection may reduce anesthesia-related risks in infants, particularly given their limited physiological reserve. The reduced cholangitis rate (29% vs. 73%) aligns with prior reports suggesting that improved hilar exposure facilitates more precise dissection of fibrotic remnants, potentially minimizing postoperative bile stasis and infection. Jaundice clearance (defined as TBIL <20 μmol/L) was achieved in 8/14 (57.1%) of the partial quadrate lobectomy group vs. 3/11 (27.3%) in the control group at 6 months (*P* = 0.025), and 10/14 (71.4%) vs. 4/11 (36.4%) at 12 months (*P* = 0.031). The lower TBIL levels (5.11 vs. 9.67 mg/dl) at 6 months suggest enhanced bile drainage efficacy, which is critical for delaying or avoiding liver transplantation in this population.

**Conclusion:**

Partial quadrate lobectomy during laparoscopic Kasai surgery reduces operation time, lowers cholangitis incidence, and improves jaundice clearance rates without increasing intraoperative blood loss or adversely affecting one-year native liver survival. It is a safe and feasible adjunct to improve early postoperative outcomes.

## 1 Background

Biliary atresia (BA) is a progressive obliterative cholangiopathy, potentially triggered by viral infections (e.g., cytomegalovirus, rotavirus) or environmental toxins such as biliatresone. Recent studies suggest that aflatoxin exposure may exacerbate disease progression in patients with GSTM1 detoxification defects, leading to accelerated hepatic fibrosis (1). The pathological hallmarks of BA include persistent inflammation and fibrosis of both intrahepatic and extrahepatic bile ducts (1, 2). Without timely surgical intervention, BA leads to irreversible liver damage, ultimately progressing to cirrhosis and liver failure, with most affected children succumbing to the disease within the first two years of life (3). The diagnosis and management of BA present significant challenges for pediatric surgeons due to the complexity and urgency of the condition.

The introduction of the Kasai procedure has dramatically enhanced the survival prospects of children with BA. With the continuous advancement of minimally invasive surgical techniques, increasing interest has emerged in investigating the feasibility, safety, and efficacy of laparoscopic Kasai surgery. Although laparoscopic techniques offer several advantages, including reduced trauma, minimized postoperative pain, and improved cosmetic outcomes, the widespread adoption of laparoscopic Kasai surgery in pediatric practice has been hindered by its high technical demands and steep learning curve (4, 5). Furthermore, the long-term efficacy of laparoscopic Kasai surgery remains a subject of ongoing debate among experts.

The complex anatomical structure of the hepatic hilum, combined with the restricted operative space, particularly in neonates, further complicates laparoscopic procedures (6, 7). In cases where biliary obstruction causes segmental liver enlargement, the difficulty in exposing the hepatic hilum increases, posing additional challenges for the surgeon, especially in younger patients with BA (8, 9). Partial Quadrate lobectomy during Kasai surgery has been proposed as a solution to this problem, as it can enlarge the surgical field, improve exposure of the hepatic hilum, and facilitate pathological assessment, which may provide valuable insights into prognosis (9). This technique selectively removes the hypertrophic anterior segment (Couinaud IVb), improving exposure of the porta hepatis under laparoscopic visualization, and enabling targeted pathological sampling of the obstructed biliary remnants. Histological evaluation of the resected IVb segment may provide insights into ductal proliferation patterns and fibrosis progression, which correlate with postoperative jaundice clearance rates. However, limited research exists on the incorporation of partial quadrate lobectomy in Kasai surgery. This study seeks to address this gap by retrospectively analyzing the early efficacy and safety of partial quadrate lobectomy in laparoscopic Kasai surgery.

## 2 Materials and methods

### 2.1 General data

This retrospective study included 25 children diagnosed with type III biliary atresia, who underwent surgical treatment in the pediatric surgery department between February 2018 and July 2022.

#### 2.1.1 Inclusion criteria

Children diagnosed with type III biliary atresia, confirmed via laparoscopic cholangiography, who subsequently underwent laparoscopic Kasai surgery.

#### 2.1.2 Exclusion criteria

1. Children with other severe congenital conditions that were deemed untreatable.
2. Children with severe cardiac or pulmonary dysfunction, or other contraindications to surgery.
3. Children who either died or were withdrawn from treatment due to unrelated diseases.

This study was approved by the regional Ethics Committee for Medical Research of Huai'an Maternal and Child Health Care Hospital Affiliated to Yangzhou University (Approval No. 2024053) and was conducted in accordance with the Declaration of Helsinki. Written informed consent to participate in the study was obtained from the parents/guardians of all patients.

In the partial quadrate lobectomy group, there were 14 patients (5 males, 9 females) with an average weight of 5.50 [4.68–6.23] kg and a mean surgical age of 66.79 ± 17.17 days. In the control group, there were 11 patients (7 males, 4 females) with an average weight of 5.50 [4.60–5.60] kg and a mean surgical age of 71.09 ± 24.32 days. There were no statistically significant differences between these baseline characteristics (*P* > 0.05). Postoperative liver pathology in all patients revealed varying degrees of fibrosis, pseudo-lobule formation, and bile thrombus formation. In some cases, intrahepatic bile duct dysplasia was also observed.

### 2.2 Surgical method

**Figure 1:**
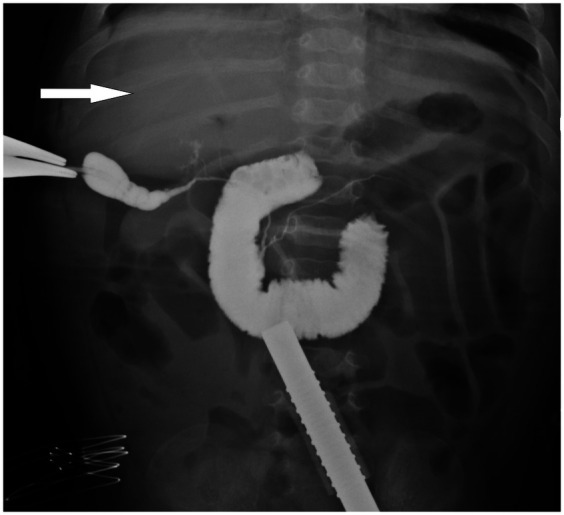
Intraoperative cholangiography image of a patient with biliary atresia. The white arrow highlights the absence of contrast filling in the intrahepatic bile ducts, indicating their non-visualization, which is characteristic of biliary atresia.

**Figure 2:**
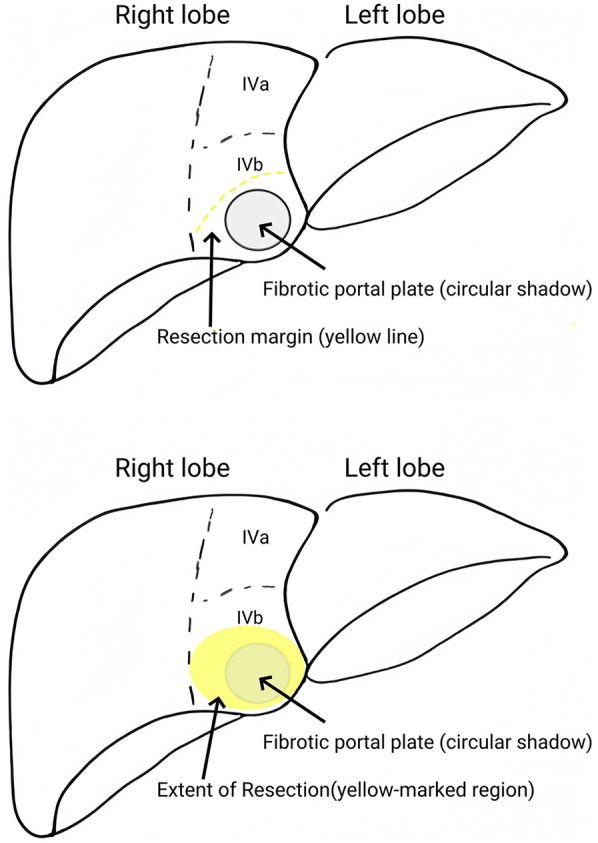
Partial quadrate lobectomy (targeting Couinaud segment IVb) was performed to optimize hilar exposure while preserving functional parenchyma (yellow-marked region).

**Figure 3:**
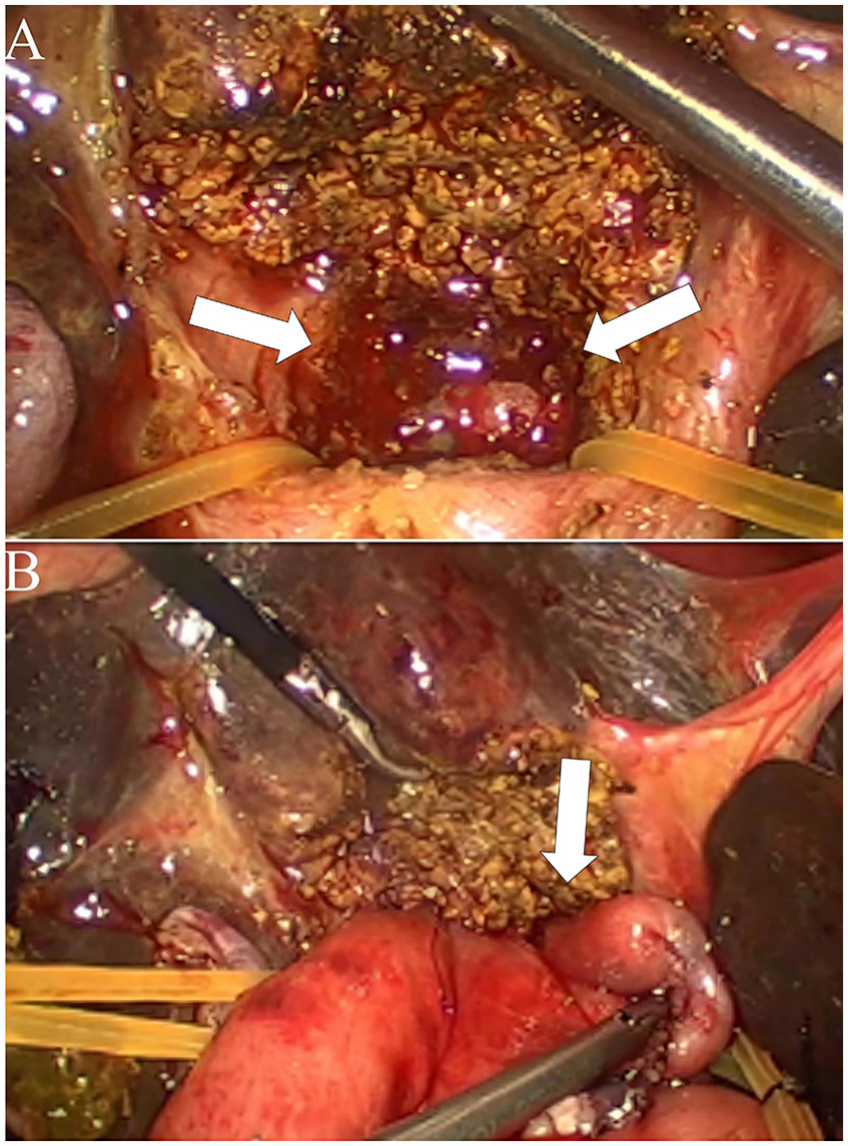
Intraoperative findings and surgical procedure for biliary atresia with partial quadrate lobectomy. **(A)** Exposure of the hepatic hilum following resection of the Partial quadrate lobectomy. The arrows indicate the dissected area of the porta hepatis, revealing the hepatic ductal structures. **(B)** Completion of hepaticoportoenterostomy after partial quadrate lobectomy. The arrow highlights the anastomosis between the hepatic ductal remnant and the roux-en-Y intestinal limb, facilitating bile drainage.

During the procedure, laparoscopic exploration of the abdominal cavity was conducted to evaluate the condition of the gallbladder (enlargement or atrophy), the extent of liver fibrosis, and the degree of hepatic hilum enlargement. Cholangiography was performed to confirm the diagnosis of type III biliary atresia (Figure 1). According to the intraoperative requirement for exposure of the hilar fibrous mass [Partial quadrate lobectomy was performed when intraoperative ultrasound confirmed: IVb segment hypertrophy (diameter ratio of IVb/right anterior portal branch >1.5); Fibrotic plate thickness >4 mm impeding safe dissection], Partial quadrate lobectomy (targeting Couinaud segment IVb)was performed to optimize hilar exposure while preserving functional parenchyma (yellow-marked region in Figure 2). The gallbladder was mobilized, and the fibrous tissue surrounding it was sutured and retracted to enhance exposure of the hepatic hilum. The fibrous masses and blood vessels of the hepatic hilum were carefully dissected and mobilized. The distal common bile duct was ligated and transected. The dissection was then extended cranially toward the hepatic hilum to further explore the fibrous masses. Both the left and right hepatic arteries were meticulously isolated, with the middle hepatic artery being identified, ligated, and transected. Dissection of the fibrous masses continued toward the liver surface. The left and right branches of the portal vein were mobilized, with elastic retraction bands applied to the left hepatic artery, left branch of the portal vein, right branch of the portal vein, and right hepatic artery, thereby enhancing exposure of the hepatic hilum. The fibrous masses at the hepatic hilum were extensively resected down to the liver surface. Following resection of the fibrous masses, bile flow was observed (Figure 3A).

**Figure 4:**
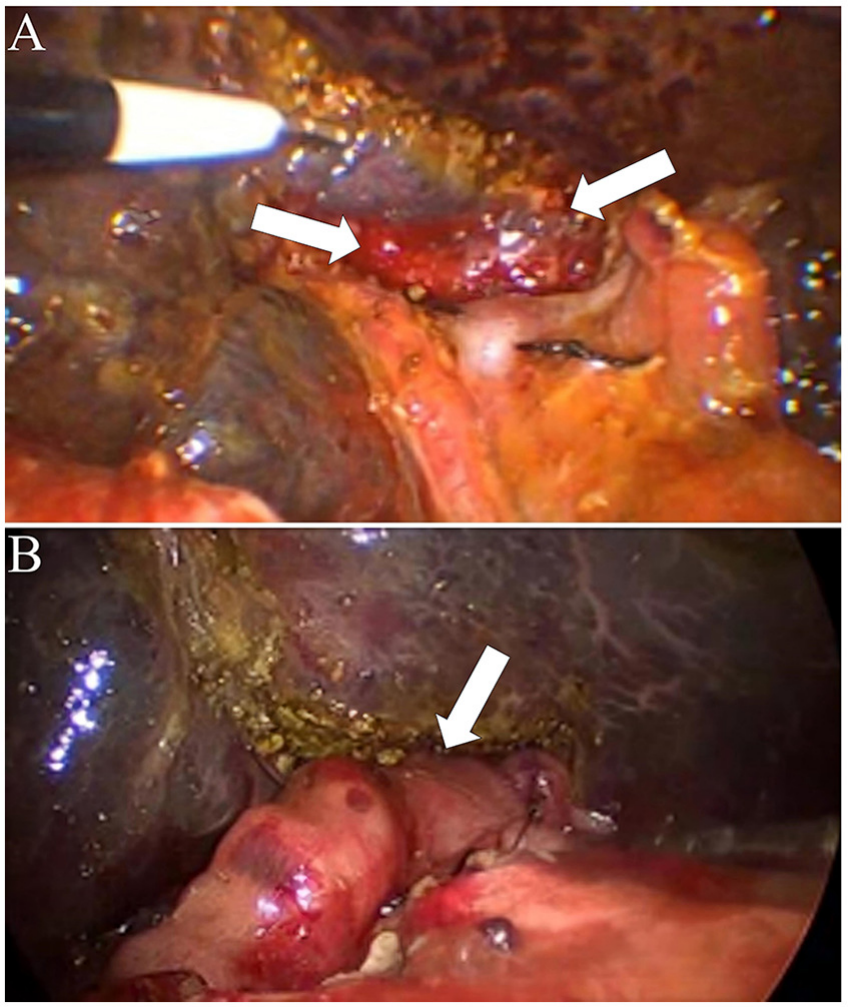
Intraoperative findings and surgical procedure for biliary atresia without partial quadrate lobectomy. **(A)** Exposure of the hepatic hilum without Partial quadrate lobectomy. The arrows indicate the porta hepatis area with the hepatic ductal structures exposed. **(B)** Completion of hepaticoportoenterostomy without partial quadrate lobectomy. The arrow highlights the anastomosis between the hepatic ductal remnant and the roux-en-Y intestinal limb, facilitating bile drainage.

The umbilical incision was extended to exteriorize a jejunal limb, which was then used to construct a Roux-en-Y hepaticojejunostomy. The jejunal limb was passed retrocolically into the abdominal cavity, and a 2-cm incision was made along the mesenteric border of the jejunum. A hepaticojejunostomy was created with an anastomotic diameter of approximately 2 cm (Figure 3B). In pediatric patients without partial resection of Couinaud segment IVb, suboptimal exposure of the hepatic portal region significantly compromises the technical feasibility of hilar enteric anastomosis (Figures 4A,B).

### 2.3 Postoperative care

Postoperative care involved continuous cardiac monitoring, fasting, gastrointestinal decompression, and intravenous nutritional support. Postoperative protocols included off-label ursodeoxycholic acid (UDCA, 10 mg/kg/day orally for 6 months) as part of historical institutional practice, though its efficacy and safety in biliary atresia remain unvalidated. Intravenous glutathione (30 mg/kg/day for 14 days) and prophylactic antibiotics (ceftriaxone 50 mg/kg/day IV for 2 weeks, followed by trimethoprim-sulfamethoxazole 2 mg/kg/day for 3 months) were administered. Glucocorticoids were discontinued after critical reassessment of their risk-benefit profile. In cases where postoperative complications such as cholangitis occurred, intravenous antibiotics and additional supportive treatments were provided as required.

### 2.4 Data processing

Statistical analyses were conducted using R version 4.3.2. Continuous data that followed a normal distribution were expressed as mean ± standard deviation, while non-normally distributed data were presented as median (interquartile range). Independent sample *t*-tests were applied to compare two groups when the data were normally distributed, while the Mann–Whitney *U*-test was used for non-normally distributed data. For multiple group comparisons, one-way ANOVA or Kruskal–Wallis (H) tests were employed as appropriate. Categorical data were described as frequencies and percentages, and comparisons between groups were made using either the chi-square test or Fisher's exact test. A two-sided *P*-value of less than 0.05 was considered statistically significant. Jaundice clearance was defined as total bilirubin (TBIL) <20 μmol/L, consistent with the criteria of Lakshminarayanan & Davenport (2016). The ULN thresholds used for conversion are based on established pediatric reference ranges: ULN thresholds: ALT (40 U/L), AST (40 U/L), GGT (50 U/L), TBA = 10 μmol/L.

## 3 Results

In the partial quadrate lobectomy group (*n* = 14), the mean age at the time of surgery was 66.79 ± 17.17 days, with an average weight of 5.50 [4.68–6.23] kg. The male-to-female ratio was 5:9. In the control group (*n* = 11), the mean surgical age was 71.09 ± 24.32 days, with an average weight of 5.50 [4.60–5.60] kg, and a male-to-female ratio of 7:4. Statistical analysis revealed no significant differences between the two groups regarding age at surgery (*P* = 0.625), gender (*P* = 0.238), or weight (*P* = 0.427) (Table 1).

**Table 1:** Descriptive table of preoperative and postoperative outcomes.

Parameter	Partial quadrate lobectomy group (*n* = 14)	Control group (*n* = 11)	Statistical significance (*P*-value)
Preoperative			
Age at surgery (days)	66.79 ± 17.17	71.09 ± 24.32	0.625
Weight (kg)	5.50 [4.68–6.23]	5.50 [4.60–5.60]	0.427
Weight *Z*-score^a^	−1.2 ± 0.8	−1.5 ± 1.1	0.321
Height *Z*-score^a^	−0.9 ± 0.6	−1.3 ± 0.9	0.218
Male:Female ratio	5:9	7:4	0.238
Average operation time	301.14 (43.69)	347.27 (26.49)	0.013
Intraoperative blood loss	20.00 [9.50, 50.00]	20.00 [9.00, 30.00]	0.517
Postoperative outcomes			
Jaundice clearance			
Cleared jaundice (TBIL <20 µmol/L)	10/14 (71.4%)	4/11 (36.4%)	0.047^b^
Improved but TBIL >4 × ULN^c^	3/14 (21.4%)	5/11 (45.5%)	0.185
Postoperative cholangitis	4 (28.6%)	8 (72.7%)	0.047
Liver failure progression			
Liver cell failure	1/14 (7.1%)	4/11 (36.4%)	0.032^b^
Liver transplantation	2 (14.29%)	2 (18.18%)	>0.999
Mortality	0/14 (0%)	2/11 (18.2%)	0.049^b^

Notes: ^a^*Z*-scores calculated using WHO growth standards.

^b^ Statistically significant (*P* < 0.05).

^c^ ULN (upper limit of normal) for TBIL: 1.17 mg/dl.

Preoperatively, the partial quadrate lobectomy group had a total bilirubin (TBIL) level of 9.40 [7.91–12.58] mg/dl, a direct bilirubin (DBIL) level of 5.52 [4.81–7.40] mg/dl, an alanine aminotransferase (ALT) 2.81 folds of ULN, and an aspartate aminotransferase (AST) 3.73 folds of ULN. In the control group, preoperative TBIL was 10.65 [9.64–11.05] mg/dl, DBIL was 5.51 [4.75–6.50] mg/dl, ALT was 1.96 folds of ULN, and AST was 3.44 folds of ULN (Table 2). There were no statistically significant differences between the two groups in terms of preoperative TBIL (*P* = 0.572), DBIL (*P* = 0.687), ALT (*P* = 0.202), or AST (*P* = 0.511). The average operation time in the partial quadrate lobectomy group was 301.14 ± 43.69 min, significantly shorter than the 347.27 ± 26.49 min in the control group (*P* = 0.013). There were no significant differences in intraoperative blood loss between the two groups (*P* = 0.517) (Table 1). Postoperative cholangitis occurred in 4/14 patients in the partial quadrate lobectomy group, compared to 8/11 in the control group, with a statistically significant difference (*P* = 0.047) (Table 1). Additionally, there were no significant differences in the one-year native liver survival or mortality rates between the two groups.

**Table 2:** Pre- and postoperative parameters.

Parameter	Group	Preoperative	6 months	12 months
TBIL (mg/dl)	Partial quadrate	9.40 [7.91–12.58]	5.11 (4.86)	2.86 (2.17)
	Control	10.65 [9.64–11.05]	9.67 (3.31)	6.77 (3.96)
DBIL (mg/dl)	Partial quadrate	5.52 [4.81–7.40]	3.79 (3.10)	2.10 (1.96)
	Control	5.51 [4.75–6.50]	7.08 (2.74)	4.62 (2.50)
ALT (×ULN)	Partial quadrate	2.81	1.73	1.57
	Control	1.96	4.44	1.11
AST (×ULN)	Partial quadrate	3.73	2.42	2.43
	Control	3.44	3.55	1.2
GGT (×ULN)	Partial quadrate	10.16	10.16	2.45
	Control	4.63	4.63	1.37

ULN thresholds: ALT (40 U/L), AST (40 U/L), GGT (50 U/L), TBA = 10 μmol/L.

In the context of the laparoscopic Kasai procedure, the implementation of partial quadrate lobectomy has been shown to significantly reduce surgical duration and diminish the incidence of cholangitis; however, it does not affect the one-year graft survival rate or contribute to an increase in mortality.

At 6 months post-operation, the total bilirubin (TBIL) levels in the partial quadrate lobectomy group were measured at 5.11 (4.86) mg/dl, while the direct bilirubin (DBIL) levels were 3.79 (3.10) mg/dl. Alanine aminotransferase (ALT) of 1.38 [0.88, 3.96] folds of ULN, aspartate aminotransferase (AST) of 2.42 [2.02, 3.52] folds of ULN, gamma-glutamyl transferase (GGT) of 8.47 [3.75, 14.17] folds of ULN, and total bile acids (TBA) of 11.42 ± 10.64 folds of ULN. In the control group, TBIL was recorded at 9.67 (3.31) mg/dl, DBIL at 7.08 (2.74) mg/dl, ALT 3.55 [1.04, 4.77] folds of ULN, AST 3.55 [2.12, 5.06] folds of ULN, GGT 3.86 [1.80, 9.45] folds of ULN, and TBA 11.57 ± 8.39 folds of ULN. Statistically significant differences were observed in TBIL and DBIL between the two groups (*P* = 0.010; *P* = 0.025). No significant differences were detected in ALT, AST, GGT, and TBA (*P* = 0.744; *P* = 0.638; *P* = 0.744; *P* = 0.973) (Table 3).

**Table 3:** Comparison of liver function tests at 6 months postoperatively.

Indicator	Total (*n* = 25)	Partial quadrate lobectomy group (*n* = 14)	Control group (*n* = 11)	Statistical significance (*P*-value)
TBIL (mg/dl)	6.85 (4.81)	5.11 (4.86)	9.67 (3.31)	0.01
DBIL (mg/dl)	5.04 (3.33)	3.79 (3.10)	7.08 (2.74)	0.025
AST (×ULN)	2.98 [2.02, 4.71]	2.42 [2.02, 3.52]	3.55 [2.12, 5.06]	0.638
ALT (×ULN)	1.92 [0.88, 4.36]	1.38 [0.88, 3.96]	3.55 [1.04, 4.77]	0.744
GGT (×ULN)	5.37 [1.82, 14.17]	8.47 [3.75, 14.17]	3.86 [1.80, 9.45]	0.744
TBA (×ULN)	11.48 ± 9.62	11.42 ± 10.64	11.57 ± 8.39	0.973

ULN thresholds: ALT (40 U/L), AST (40 U/L), GGT (50 U/L), TBA = 10 μmol/L.

At 12 months post-operation, TBIL in the partial quadrate lobectomy group was 2.86 (2.17) mg/dl, DBIL was 2.10 (1.97) mg/dl, alanine aminotransferase (ALT) 1.57 [0.93, 2.43] folds of ULN, aspartate aminotransferase (AST) 2.43 [1.51, 3.04] folds of ULN, gamma-glutamyl transferase (GGT) 2.45 [0.71, 6.23] folds of ULN, and total bile acids (TBA) 6.95 [3.49, 8.56] folds of ULN. In the control group, TBIL was 6.78 (3.96) mg/dl, DBIL was 4.62 (2.50) mg/dl, ALT 1.11 [0.75, 2.41] folds of ULN, AST 1.20 [1.02, 2.73] folds of ULN, GGT 1.37 [0.58, 3.11] folds of ULN, and TBA 4.44 [1.88, 7.43] folds of ULN. Statistically significant differences were identified in TBIL and DBIL between the two groups (*P* = 0.043; *P* = 0.025). No significant differences were noted in ALT, AST, GGT, and TBA (*P* = 0.669; *P* = 0.417; *P* = 0.536; *P* = 0.740) (Table 4).

**Table 4:** Comparison of liver function tests at 12 months postoperatively.

Indicator	Total (*n* = 25)	Partial quadrate lobectomy group (*n* = 14)	Control group (*n* = 11)	Statistical significance (*P*-value)
TBIL (mg/dl)	4.47 (3.53)	2.86 (2.17)	6.78 (3.96)	0.043
DBIL (mg/dl)	3.14 (2.48)	2.10 (1.97)	4.62 (2.50)	0.025
AST (×ULN)	1.73 [1.10, 3.18]	2.43 [1.51, 3.04]	1.20 [1.02, 2.73]	0.417
ALT (×ULN)	1.23 [0.78, 2.63]	1.57 [0.93, 2.43]	1.11 [0.75, 2.41]	0.669
GGT (×ULN)	1.84 [0.60, 5.12]	2.45 [0.71, 6.23]	1.37 [0.58, 3.11]	0.536
TBA (×ULN)	5.28 [2.60, 8.61]	6.95 [3.49, 8.56]	4.44 [1.88, 7.43]	0.74

ULN thresholds: ALT (40 U/L), AST (40 U/L), GGT (50 U/L), TBA = 10 μmol/L.

In conclusion, the implementation of partial quadrate lobectomy during the laparoscopic Kasai procedure is associated with an improved jaundice clearance rate in pediatric patients post-operation.

At 12 months, Doppler ultrasound revealed normal portal venous flow velocities (28 ± 5 cm/s) in both groups, with no cases of portal vein thrombosis. Mild perihepatic lymphadenopathy (<1 cm) was observed in 3/14 (21.4%) patients in the partial quadrate lobectomy group vs. 5/11 (45.5%) controls (*p* = 0.08). No biliary strictures or intrahepatic calculi were detected.

## 4 Discussion

The only definitive treatment for biliary atresia is surgical intervention, with the Kasai procedure (hepatic portoenterostomy) being the primary surgical technique. This procedure alleviates bile duct obstruction by reconstructing the bile drainage pathway (10, 11). However, despite successful Kasai surgery, factors such as autoimmunity, ongoing inflammation, and fibrosis of the bile ducts may continue to drive disease progression, leading to cirrhosis or liver failure in some cases (12, 13). As medical technology advances, so do diagnostic and therapeutic approaches to biliary atresia, along with surgical techniques and postoperative care. Current literature reports a 2-year native liver survival rate of approximately 50%–60% following Kasai surgery (14, 15). In the absence of liver transplantation, about 60% of children who undergo timely Kasai surgery are expected to reach adulthood (14). Thus, although liver transplantation is considered the definitive treatment for biliary atresia in certain regions, the Kasai procedure remains the primary and most widely used treatment (16). It is widely recognized that the surgical experience of the operating surgeon significantly impacts the prognosis of children with biliary atresia (17), and there is an ongoing need to refine and improve surgical techniques in pediatric surgery (18).

In 2002, Esteves et al. (19) were the first to report two cases of laparoscopic Kasai surgery. Since then, numerous scholars have documented similar cases of laparoscopic Kasai surgery (20–22). However, whether laparoscopic Kasai surgery can achieve equivalent or superior outcomes compared to open Kasai surgery remains a subject of debate. In 2015, Japanese scholar Murase et al. (23) reported that the jaundice clearance rate following laparoscopic Kasai surgery reached 83.3%, significantly higher than the 50% clearance rate observed in the open surgery group. Since 2012, our hospital has been investigating laparoscopic surgery. In this study, the overall jaundice clearance rate at six months postoperatively, for 25 laparoscopic Kasai surgeries performed between February 2018 and July 2022, was 68.7%, comparable to data from other large-scale medical centers in China. Liver regeneration was assessed via contrast-enhanced CT volumetry at 6 months postoperatively. Segmental hypertrophy of the adjacent Couinaud segments (III and V) compensated for 89.2 ± 6.5% of the resected IVb volume, with no evidence of fibrotic scarring on elastography (shear-wave velocity: 1.2 ± 0.3 m/s, within normal limits).

Kasai surgery has consistently been one of the most challenging procedures in pediatric surgery. The steps include gallbladder resection, exposure of the hepatic hilum, dissection of the hepatic hilum, removal of fibrous masses from the hepatic hilum, preparation of the Roux-en-Y jejunal loop, and hepaticojejunostomy of the bile ducts at the hepatic hilum. These steps involve multiple anatomical layers and considerable complexity, frequently resulting in prolonged surgical times (24, 25). Couinaud segment IVb resection of the hypertrophic visceral portion optimizes caudal exposure of the hilar fibrotic plate, enabling complete removal of fibrotic portal plate while minimizing parenchymal injury through a limited resection scope. In this study, the shorter operative time (301 vs. 347 min) associated with partial quadrate lobe resection may reduce anesthesia-related risks in infants, particularly given their limited physiological reserve (15). After partial quadrate lobectomy, the surgical duration was notably reduced. Furthermore, in some early cases, the immature partial quadrate lobectomy technique contributed to longer operation times. This indicates that, although the additional surgical steps did not significantly affect the overall surgical duration, they ultimately shortened the procedure time.

Theoretically, partial quadrate lobectomy requires cautious manipulation and meticulous hemostasis, which might otherwise extend the surgical duration. However, in cases where significant liver enlargement is present, partial quadrate lobectomy allows for clearer exposure of the hepatic hilum's anatomical layers, thereby facilitating more precise and efficient execution of the remaining surgical steps (8, 9). As a result, the time saved during other parts of the procedure compensates for the additional step of partial quadrate lobectomy, ultimately shortening the total surgery time.

Due to the liver's rich blood supply, insufficient hemostasis during resection could lead to substantial blood loss. In laparoscopic surgery, excessive intraoperative bleeding can impair the clarity of the surgical field and increase the need for transfusion. Apart from surgery duration, an important question is whether partial quadrate lobectomy in laparoscopic Kasai surgery increases intraoperative blood loss. While no similar studies have specifically addressed this issue, previous literature and clinical experience suggest that the segmental liver lacks critical anatomical structures, making resection relatively straightforward (26, 27).

In this study, the partial quadrate lobectomy group exhibited slightly higher blood loss than the control group, although the difference was not statistically significant (*P* = 0.517), and this variation had no clinical impact. Multiple factors, such as the child's coagulation function, anatomical variations, and the surgeon's experience, could influence intraoperative blood loss. As long as the branches of the left portal vein are carefully handled to avoid injury, partial quadrate lobectomy can be performed with minimal blood loss, eliminating the need for portal vein clamping during surgery (8). Among the 14 children in the partial quadrate lobectomy group, only one required transfusion due to preoperative coagulation dysfunction. Postoperative liver pathology revealed varying degrees of fibrosis, pseudo-lobule formation, and bile thrombus formation in all 14 children; some cases also exhibited intrahepatic bile duct dysplasia. The three children who died in the partial quadrate lobectomy group were diagnosed with intrahepatic bile duct dysplasia. Liver pathology can be directly obtained from the resected liver segment, which eliminates the need for biopsy of normal liver tissue during surgery, offering significant prognostic value. The single mortality in the partial quadrate lobectomy group (Case 10) occurred in a patient with ductal plate malformation on histology, highlighting that jaundice clearance does not preclude progression of congenital ductal dysmorphogenesis, as described in recent genomic studies (1).

The primary objective of Kasai surgery is to relieve bile duct obstruction, eliminate jaundice, and ensure bile drainage. Blood bilirubin levels are the most direct indicator of whether Kasai surgery has successfully achieved bile drainage and are important markers of liver function (28). Recent studies suggest that aflatoxin exposure may exacerbate disease progression in patients with GSTM1 detoxification defects, leading to accelerated hepatic fibrosis (13, 15). Segmental liver resection may reduce toxin load by removing diseased parenchyma harboring toxin-induced damage (13). In this study, the total bilirubin (TBIL) and direct bilirubin (DBIL) levels in both groups decreased significantly at various postoperative time points compared to preoperative levels, indicating successful restoration of bile flow in both groups. Further analysis revealed that at seven days postoperatively, the TBIL and DBIL levels in the partial quadrate lobectomy group were higher than in the control group, though the difference was not statistically significant. These results suggest that partial quadrate lobectomy may have a short-term impact on bilirubin metabolism. However, at six and 12 months postoperatively, TBIL and DBIL levels in the partial quadrate lobectomy group had significantly decreased compared to preoperative levels (6 months: *P* = 0.010, *P* = 0.025; 12 months: *P* = 0.043, *P* = 0.025), while other liver function indicators showed no significant differences. These findings suggest that partial quadrate lobectomy can effectively improve jaundice clearance without exacerbating liver dysfunction, as long as the liver can compensate. While UDCA was empirically used in our cohort based on historical institutional protocols, we acknowledge the absence of RCT evidence supporting its efficacy in biliary atresia. Emerging data suggest potential hepatotoxicity from UDCA-derived lithocholic acid, particularly in pediatric populations (29, 30). This underscores the need for rigorous pharmacovigilance and biomarker monitoring in future studies (29, 30).

A key factor in the success of Kasai surgery is ensuring maximum bile drainage through the anastomosis. The quality of the anastomosis and postoperative bile drainage are crucial factors influencing the volume of bile flow over time. In children with biliary atresia, bile accumulation may enlarge the liver, affecting surgical field exposure, and inflammation and fibrosis may narrow the bile ducts, both of which compromise anastomotic quality (15). Based on our experience, the benefits of partial quadrate lobectomy for anastomosis quality can be summarized as follows:

It allows for more thorough exposure of the hepatic hilum. The magnification provided by the laparoscope enables the surgeon to select an optimal anastomosis site.

It facilitates better exposure of the left and right branches of the portal vein, allowing for precise shaping or anastomosis of the narrow hepatic hilum (9).

Removal of the enlarged segmental liver relieves pressure on the high-position anastomosis and increases bile drainage.

Beyond its impact on surgery duration, intraoperative blood loss, and jaundice clearance rates, we believe that the advantages offered by partial quadrate lobectomy may theoretically reduce long-term postoperative complications, such as cholangitis and anastomotic strictures, in children with biliary atresia. In this study, the partial quadrate lobectomy group had significantly shorter surgery times, lower cholangitis incidence, and better jaundice clearance rates compared to the control group, suggesting that partial quadrate lobectomy contributes to more satisfactory anastomosis outcomes. However, due to the relatively small sample size, further research with larger sample sizes and longer follow-up periods is needed to confirm these findings.

## 5 Limitations subsection

This study did not evaluate lithocholic acid levels or long-term UDCA safety outcomes. The off-label use of UDCA in children with biliary atresia remains controversial, as highlighted by Bezerra et al. (2018), who emphasized the lack of controlled trials to guide clinical practice. Future research must prioritize RCTs to establish evidence-based postoperative medical management.

## Data Availability

The original contributions presented in the study are included in the article/Supplementary Material, further inquiries can be directed to the corresponding authors.
