# Corrigendum: partial quadrate lobectomy improves early outcomes of laparoscopic Kasai surgery in type III biliary atresia

**DOI:** 10.3389/fped.2025.1631865

**Published:** 2025-06-17

**Authors:** Chunhui Gu, Jian Sun, Lihong Ding, Bing Li, Youcheng Zhang, Guoqing Jiang

**Affiliations:** ^1^ Department of Pediatric Surgery, Huai’an maternal and child health care hospital affiliated to yangzhou university, Huai’an, China; ^2^ Department of Hepatobiliary Surgery, Northern Jiangsu People’s Hospital, Yangzhou, China; ^3^ Department of Hepatobiliary Surgery, Northern Jiangsu People’s Hospital Affiliated to Yangzhou University, Yangzhou, Jiangsu, China; ^4^ Nanjing University of Chinese Medicine, Nanjing, China

**Keywords:** laparoscopic Kasai, biliary atresia, partial quadrate lobectomy, segmentectomy, postoperative outcomes

A Corrigendum on Partial quadrate lobectomy improves early outcomes of laparoscopic Kasai surgery in type III biliary atresia By Gu C, Sun J, Ding L, Li B, Jiang G and Zhang Y (2025). Front. Pediatr. 13:1541455. doi: 10.3389/fped.2025.1541455

In the published article, there was an error in the affiliations. Instead of

“1 Department of Pediatric Surgery, Huai’an Maternal and Child Health Care Hospital Affiliated to Yangzhou University, Huai’an, China

2 College of Pharmacy, Nanjing University of Chinese Medicine, Nanjing, China

3 Department of Hepatobiliary, Northern Jiangsu People's Hospital Affiliated to Yangzhou University, Yangzhou, China”

It should be:

“^1^ Department of Pediatric Surgery, Huai’an Maternal and Child Health Care Hospital Affiliated to Yangzhou University, Huai’an, China

^2^ Department of Hepatobiliary Surgery, Northern Jiangsu People's Hospital, Yangzhou, China

^3^ Department of Hepatobiliary Surgery, Northern Jiangsu People's Hospital Affiliated to Yangzhou University, Yangzhou, Jiangsu, China

^4^ Nanjing University of Chinese Medicine, Nanjing, China”

In the published article, there was an error regarding the affiliations linked to Chunhui Gu and Guoqing Jiang. Chunhui Gu should be linked to the affiliations 1, 2, and 3. Guoqing Jiang should be linked to affiliations 2 and 3.

In the published article, there was an error in the order of the author list. The corrected order of author list appears below.

Chunhui Gu1,2,3 †, Jian Sun1 †, Lihong Ding 1,4, Bing Li 1, Youcheng Zhang 1*, Guoqing Jiang 2,3*

The authors apologize for these errors and state that this does not change the scientific conclusions of the article in any way. The original article has been updated.

